# To: Identification of distinct phenotypes and improving prognosis using metabolic biomarkers in COVID-19 patients

**DOI:** 10.62675/2965-2774.20250279

**Published:** 2025-05-28

**Authors:** Carla Alexandra Scorza, Fulvio Alexandre Scorza, Josef Finsterer

**Affiliations:** 1 Universidade Federal de São Paulo Escola Paulista de Medicina São Paulo SP Brasil Escola Paulista de Medicina, Universidade Federal de São Paulo - São Paulo (SP), Brasil.; 2 Neurology & Neurophysiology Center Vienna Austria Neurology & Neurophysiology Center - Vienna, Austria.

To the Editor

We were interested in reading the article by Santana et al. on the accuracy of specific serum biomarkers in predicting mortality in severe acute respiratory syndrome coronavirus 2 (SARS-CoV-2) infection^(1)^ It was found that cortisol, resistin, leptin, insulin, and ghrelin levels differed between World Health Organization (WHO) severity groups and that lower ghrelin and higher cortisol levels were associated with mortality.^(1)^ Two different phenotypes were associated with disease severity but not mortality.^(1)^ It was concluded that adding biomarkers to the clinical predictors of mortality significantly improved the accuracy in determining the prognosis of SARS-CoV-2.^(1)^ The study is impressive, but some points should be discussed.

The first point is that the prognosis of disease progression and mortality may depend not only on biomarkers such as cortisol, resistin, leptin, insulin, and ghrelin but rather on the severity of the infection, the extent and severity of multi-organ involvement, the response to treatment, the immunocompetence of the person affected, comorbidities, concomitant medications, vaccination status, the coping strategies available to the person affected and the social network available to the patient.^(2)^ It may also depend on adapting to entirely new and previously unexperienced situations. The outcome of SARS-CoV-2 can also depend heavily on the strain of the virus, the response to antiviral treatment, and whether the patient requires mechanical ventilation, extracorporeal membrane oxygenation (ECMO), or has superinfections, including sepsis. Surprisingly, obese or overweight patients may have lower mortality and higher survival rates.^(3)^

The second point is that only cortisol was an independent predictor of mortality.^(1)^ Since cortisol has many different effects, such as suppressing and blocking acquired and inherited inflammatory and immunological responses by blocking the transcription factor NF-κB and increasing the synthesis of annexin A1 or the production and distribution of neutrophils, granulocytes, erythrocytes, and platelets (increases the number of blood cells), it could be a sign of an intact and appropriate response to the SARS-CoV-2 rather than a sign of morality. How can we explain that increased cortisol levels predict mortality?

A third point is that a positive reverse transcriptase polymerase chain reaction (RT-PCR) and positive antigen tests diagnosed COVID-19. Since the antigen test has a lower sensitivity,^(4)^ the diagnosis of SARS-CoV-2 by an antigen test may not be as reliable as by a PCR test, so the latter should be preferred over an antigen test. In how many of the included patients was SARS-CoV-2 diagnosed using an antigen test, and in how many by using RT-PCR?

A fourth point is that the classification of the severity of COVID-19 infection based solely on the WHO classification can be misleading, as this only assesses lung involvement. However, SARS-CoV-2 infection may not always initially manifest itself in the lungs but also in several other organs, e.g. hypogeusia, hyposmia, non-specific abdominal symptoms, corneal congestion, deep vein thrombosis and less commonly as meningitis, delirium, acute disseminated encephalomyelitis, epilepsy, headache, transverse myelitis, Guillain-Barre syndrome, facial paralysis, myositis, rhabdomyolysis, conjunctivitis, uveitis, carotid artery occlusion or skin lesions.^(5)^ Therefore, we should know whether patients with extra-pulmonary initial symptoms and signs were also included or whether only COVID-19 patients with only pulmonary disease were included.

The excellent study has limitations that should be addressed before final conclusions are drawn. Clarification of the weaknesses would strengthen the conclusions and could improve the study. Clinical rather than laboratory parameters can more reliably predict mortality in SARS-CoV-2 infections.
